# Mirror Electromyografic Activity in the Upper and Lower Extremity: A Comparison between Endurance Athletes and Non-Athletes

**DOI:** 10.3389/fnhum.2017.00485

**Published:** 2017-09-29

**Authors:** Tom Maudrich, Rouven Kenville, Jöran Lepsien, Arno Villringer, Patrick Ragert, Christopher J. Steele

**Affiliations:** ^1^Institute for General Kinesiology and Exercise Science, Faculty of Sport Science, University of Leipzig, Leipzig, Germany; ^2^Department of Neurology, Max Planck Institute for Human Cognitive and Brain Sciences, Leipzig, Germany; ^3^Clinic for Cognitive Neurology, University of Leipzig, Leipzig, Germany; ^4^Douglas Mental Health University Institute, McGill University, Montreal, QC, Canada

**Keywords:** mirror activity, motor overflow, neuroplasticity, sports, endurance exercise

## Abstract

During unimanual motor tasks, muscle activity may not be restricted to the contracting muscle, but rather occurs involuntarily in the contralateral resting limb, even in healthy individuals. This phenomenon has been referred to as mirror electromyographic activity (MEMG). To date, the physiological (non-pathological) form of MEMG has been observed predominately in upper extremities (UE), while remaining sparsely described in lower extremities (LE). Accordingly, evidence regarding the underlying mechanisms and modulation capability of MEMG, i.e., the extent of MEMG in dependency of exerted force during unilateral isometric contractions are insufficiently investigated in terms of LE. Furthermore, it still remains elusive if and how MEMG is affected by long-term exercise training. Here, we provide novel quantitative evidence for physiological MEMG in homologous muscles of LE (tibialis anterior (TA), rectus femoris (RF)) during submaximal unilateral dorsiflexion in healthy young adults. Furthermore, endurance athletes (EA, *n* = 11) show a higher extent of MEMG in LE compared to non-athletes (NA, *n* = 11) at high force demands (80% MVC, maximum voluntary contraction). While the underlying neurophysiological mechanisms of MEMG still remain elusive, our study indicates, at least indirectly, that sport-related long-term training might affect the amount of MEMG during strong isometric contractions specifically in trained limbs. To support this assumption of exercise-induced limb-specific MEMG modulation, future studies including different sports disciplines with contrasting movement patterns and parameters should additionally be performed.

## Introduction

During unimanual motor tasks, muscle activity may not be restricted to the contracting muscle, but has also been reported to occur involuntarily in the contralateral resting limb. This phenomenon has been referred to as mirror electromyographic (EMG) activity (Sehm et al., 2010, 2016). Mirror electromyographic activity (MEMG) has been observed during the performance of simple and complex motor tasks (Uttner et al., 2007), especially during strong unimanual voluntary contractions in healthy individuals (Zijdewind and Kernell, 2001; van Duinen et al., 2008; Sehm et al., 2010, 2016) and in patients suffering from neurological diseases such as Parkinson’s disease (Cincotta et al., 2006; Ottaviani et al., 2008). MEMG is thought to be the evolutionary remnant of the so called *basic-mirror-movement-mode* of the central nervous system (CNS) following an ontogenetic learning process to decouple both hands and enable independent hand movements (Uttner et al., 2007).

Recent research provided evidence that MEMG increases as a function of force demands during unimanual contractions of hand muscles, which in turn is associated with functional alterations in sensorimotor networks as assessed with functional magnetic resonance imaging (fMRI; Sehm et al., 2010). This observation is in accordance with the concept of motor overflow, which describes the occurrence of involuntary homologous muscle activity during unilateral movements (Yensen, 1965). It is assumed that this phenomenon is due to ongoing modulations of interhemispheric communication during unilateral contractions with progressively higher force demands, resulting in interhemispheric facilitation (IHF), which in turn leads to bilateral activation of motor-relevant brain regions (Perez and Cohen, 2008; Sehm et al., 2016). Recent findings have provided further evidence for bilateral activation of homologous muscle representation within M1 during unilateral movements as a result of IHF, quantified by transcranial magnetic stimulation (TMS; Chiou et al., 2013a, 2014).

To date, the occurrence of the physiological (non-pathological) form of MEMG is sparsely characterized in lower extremities (LE) of healthy adults during the performance of unilateral isometric leg contractions yet, there is some first evidence for contralateral muscle co-contraction during unilateral ankle dorsiflexion (Dimitrijevic et al., 1992). However, this investigation did not systematically quantify the amount of involuntarily ocurring muscle activity, because it was not the outcome measure of interest. Additionally, the observed contralateral co-contraction of homologous muscles in this study was not robust across subjects and submaximal force levels. Furthermore, it still remains elusive if and how MEMG is affected by long-term exercise training. Sports participation induces structural and functional neural changes as a result of neuroplastic mechanisms including the integrity of major white matter tracts as well as the modulation of interhemispheric communication between motor-relevant brain regions (Schlaffke et al., 2014; Svatkova et al., 2015). Therefore, it is tempting to speculate that long-term sports training might be able to modulate underlying mechanisms of MEMG, which subsequently could lead to observable behavioral adaptations during unilateral isometric contractions of hand and leg muscles.

Here we investigated the influence of long-term endurance sports expertise on the extent of MEMG in healthy young adults. We first hypothesized that MEMG is not restricted to upper extremities (UE) but can also be reliably observed in LE of healthy adults and further, that the extent of MEMG is positively related to the amount of force produced during submaximal unilateral motor tasks. In addition, we expected that endurance athletes (EA) show more MEMG compared to normal participants during strong unilateral contractions of hand and leg muscles due to their modulated neuroplastic properties of motor-relevant brain areas induced by regular physical exercise (Schlaffke et al., 2014; Svatkova et al., 2015). This study aims to provide new insights into the phenomenon of MEMG, especially in terms of LE during isometric contractions with different force requirements, as well as its modulation capability through long-term endurance sports.

## Materials and Methods

### Ethical Approval

The study was approved by the local ethics-committee of the Medical Faculty at the University of Leipzig (ref.-nr. 429-15-16112015). All subjects gave written informed consent to participate in the experiments according to the Declaration of Helsinki, and were compensated for participation.

### Subjects

In order to statistically confirm our explorative main hypothesis of MEMG in LE, we performed an* a priori* sample size estimation, based on previous results of MEMG-investigations in UE during strong unilateral contractions (Sehm et al., 2010, 2016). Because we aimed to test a one tailed hypothesis (increasing MEMG with higher force demands compared to muscles at rest), the normal distribution probability value for a 95%-confidence interval of *Z* = 1.64 was chosen (Sullivan, 2011). The estimated sample size to obtain sufficient test power was *n* = 17. In the present study, a total of 22 healthy, male, young adults were recruited from the local Max-Planck-Institute database as well as through public advertisement. Inclusion criteria for EA consisted of an individual training history of at least 2 years and regular practice in their respective sports discipline of at least 8 h per week. Non-athletes (NA) were not allowed to do more than 2 h of combined sports activities (any specific physical activity outside of their daily routine) per week. The investigated sample of this study consisted of EA (median (interquartile range (IQR), *n* = 11; age: 23.0 (6.0) years; bodyweight: 70.0 (7.0) kg)) and NA (*n* = 11; age: 25.0 (7.0) years; bodyweight: 76.0 (14.0) kg). EA trained and performed in the following endurance sports disciplines: triathlon, long-distance running, cycling. On average, EA trained 13.0 (5.0) h/week and had been training for 84.0 (36) months in their respective disciplines. On the other hand, NA performed an average of less than 1 h of combined sports activities (any specific physical activity outside of their daily routine) per week (0.0 (2.0) h/week). Additionally, participants (either EA or NA) with regular practice of musical instruments were excluded from participation in this study. This was motivated by the fact that recent studies have shown that musical training induces functional and structural plasticity in the corpus callosum (Steele et al., 2013; Vollmann et al., 2014) which in turn might affect the amount of MEMG. According to the Oldfield handedness inventory (Oldfield, 1971) all participants were right handed (laterality quotient: 87.0 (20.0)). Group comparisons of age, bodyweight, LQ and the amount of weekly sports activities between EA and NA were performed using non-parametric Mann-Whitney-U-test since the data was not normally distributed.

### Procedure

Participants performed a behavioral task either for the UE or LE on two separate sessions in randomized order. Between each session there was a rest period of at least 1 week in order to avoid task-related impacts of cognitive or muscular fatigue. During both sessions we acquired bilateral EMG recordings of a proximal and distal muscle of the UE and LE respectively (see “EMG Recordings” Section for further details). Participants were instructed to avoid alcohol and caffeine 24 h prior to each session because of their well-known influences on motor control and CNS functioning (Pesta et al., 2013).

### Motor Tasks

**Figure 1:**
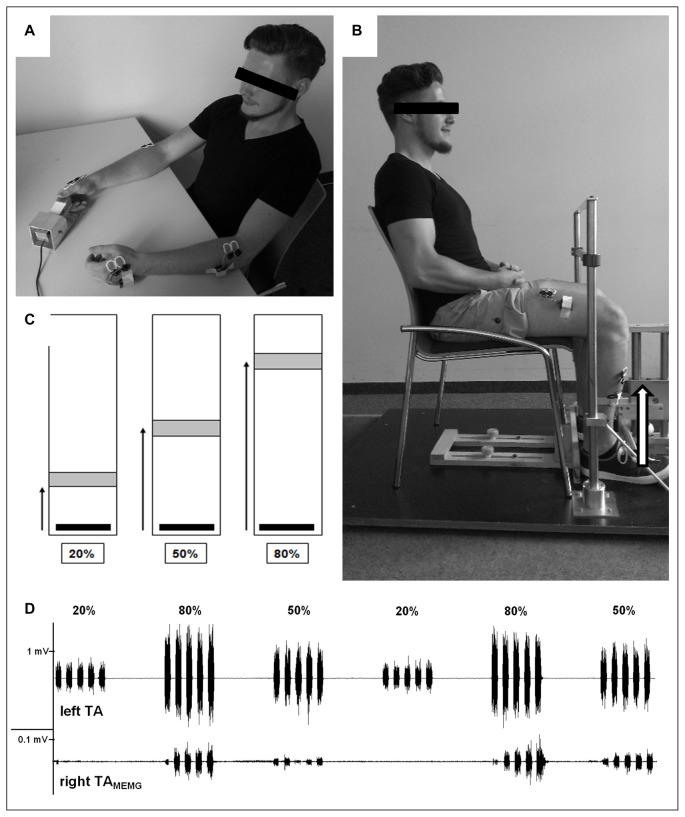
Experimental design. **(A)** Demonstration of upper extremity (UE)-testing. With one hand participants operated a custom made force sensor to perform an isometric pinch force task using their thumb and index finger. Participants were instructed to rest and relax their inactive hand on the table. EMG was recorded from bilateral first dorsal interossei (FDI) and brachioradialis (BR) during unimanual contrations. **(B)** Demonstration of lower extremity (LE)-testing. Participants had to operate a custom-made force sensor by lifting one foot with the set heel (dorsiflexion) against resistance while the other foot was resting. Both hands were placed on their lap and should not be moved throughout LE testing. EMG was recorded from bilateral tibialis anterior (TA) and rectus femoris (RF) during unilateral contractions. **(C)** Visual feedback provided to all participants during testing of both UE and LE where they had to move and hold (3 s) a vertical cursor (black bar) into a stationary target field (gray box) as fast and accuratly as possible. The target field and the force required to reach it was adjusted to each individual’s maximum voluntary contraction (MVC; i.e., greater distances required higher levels of force generation). **(D)** Representative EMG-recordings of voluntary muscle activity of left TA (upper trace) and subliminal involuntary mirror electromyographic (MEMG) in right TA (lower trace) during unilateral contractions with differing force requirements (20%, 50%, 80% MVC). Note the different scaling of both traces. In this example traces, increasing force demands resulted in higher amounts of MEMG. Please note that the participant displayed in **(A)** and **(B)** gave written informed consent to use the pictures illustrating the study design.

During each **UE testing** session, participants were seated upright and comfortably in a chair with their forearms resting on a table in front of them. With one hand they operated a custom made force sensor to perform an isometric pinch force task using their thumb and index finger. Participants were instructed to rest and relax their inactive hand on the table and to focus their attention on the active hand (see Figure 1A). Beside that, they did not receive any further visual or auditory feedback about ongoing MEMG throughout the experiment in order to avoid intentional inhibition of involuntarily occurring muscle activity. Prior to UE testing, participants had to exert their maximal voluntary force three times (trial 1–3) for a duration of 3 s with a rest period of 1 min between each trial. To ensure truly maximal effort, participants were verbally encouraged by the researcher in accordance to a standardized protocol. Subsequently, the respective trials were averaged and defined as the individual maximum voluntary contraction (MVC).

For the following **UE testing**, participants received visual feedback on a standard PC monitor using Presentation 16.5 (NeuroBehavioral Systems, Albany, NY, USA) where they had to move a vertical cursor into a stationary target field as quickly and accurately as possible. The target field and the force required to reach it were adjusted to each individual’s MVC (i.e., greater distances required higher levels of force generation, see Figure 1C). The applied isometric force was sampled digitally at 800 Hz and displayed on the PC screen at 60 Hz. UE testing consisted of a block design with varying force levels adjusted to individual MVC (20%, 50%, 80% MVC). One block consisted of five isometric contractions of one force level, with each contraction lasting 3 s and followed by a 3 s rest period. A single block lasted 30 s and was followed by an additional 30 s resting period. Each participant performed five blocks of each force level for a total of 15 blocks. Hence, time to complete UE testing of one hand was 15 min. The order of the force blocks was pseudo randomized to eliminate any systematic effects of muscular fatigue on MEMG. After another rest period of approximately 5 min, participants performed the identical procedure with the other hand. Hand order was also randomized across participants.

**LE testing** followed an identical procedure to that described above, but was performed on a separate day. Here, participants were seated in a chair with their hip and knee joints at a right angle and the upper body upright. They operated a custom-made force sensor by lifting one foot from the toes while the heel was set on the ground (dorsiflexion) against resistance while the other foot was resting. Both hands were placed on their lap and were not moved throughout testing. Participants were instructed to concentrate on moving their ankle joints to complete the isometric force task without lifting their thighs (see Figure 1B). As with the UE procedure described above, legs were tested consecutively in a randomized order across participants.

### EMG Recordings

EMG recordings during motor performance were performed using a wireless Desktop Direct Transmission System (NORAXON Inc., Scottsdale, AZ, USA). Bipolar surface electrodes (Ag/AgCl; diameter: 1 cm) were attached bilaterally on a distal (UE: first dorsal interossei (FDI), LE: tibialis anterior (TA)) and proximal muscle (UE: brachioradialis (BR), LE: rectus femoris (RF)) depending on UE/LE testing procedures.

The distal muscles (FDI and TA) were the primary sites of interest, while MEMG from the proximal muscles (BR and RF) was included as secondary outcome measures (for results see Table 1). Inter-electrode distance was standardized by the electrode diameter at 2 cm and electrodes were placed in parallel orientation relative to the muscle fibers. The bilateral set-up allowed us to measure EMG activity over the primary moving muscles as well as subliminal MEMG over the homologous muscles of the resting limb during UE/LE testing (see Figure 1D for details). Data was recorded with a sample frequency of 1500 Hz, input impedance > 100 MOhm, Common Mode Rejection Ratio (CMRR) > 100 dB and a gain of 500.

**Table 1:** Voluntary and involuntary mean EMG activity of all tested force levels (20%, 50%, 80% MVC).

		**Left**	**Left**	**Left**	**Right**	**Right**	**Right**	**Right**	**Right**	**Right**
		FDI_20%*_	FDI_50%*_	FDI_80%*_	**FDI_MEMG20%†_**	**FDI_MEMG50%†_**	**FDI_MEMG80%†_**	**BR_MEMG20%†_**	**BR_MEMG50%†_**	**BR_MEMG80%†_**
	EA	0.16 (0.13)	0.46 (0.30)	0.79 (0.44)	1.07 (0.27)	1.68 (0.93)	2.24 (1.64)	1.04 (0.08)	1.14 (0.11)	1.37 (0.53)
	NA	0.16 (0.10)	0.48 (0.28)	0.84 (0.22)	1.04 (0.06)	1.21 (0.31)	1.63 (0.78)	1.02 (0.02)	1.07 (0.13)	1.26 (0.48)
**UE**		**Right**	**Right**	**Right**	**Left**	**Left**	**Left**	**Left**	**Left**	**Left**
		FDI_20%*_	FDI_50%*_	FDI_80%*_	**FDI_MEMG20%†_**	**FDI_MEMG50%†_**	**FDI_MEMG80%†_**	**BR_MEMG20%†_**	**BR_MEMG50%†_**	**BR_MEMG80%†_**
	EA	0.15 (0.18)	0.40 (0.29)	0.65 (0.21)	1.06 (0.16)	1.40 (0.62)	1.89 (1.57)	1.02 (0.08)	1.08 (0.14)	1.25 (1.00)
	NA	0.18 (0.08)	0.49 (0.35)	0.84 (0.42)	1.00 (0.10)	1.09 (0.57)	1.54 (0.83)	1.02 (0.04)	1.06 (0.15)	1.20 (0.31)
		**Left**	**Left**	**Left**	**Right**	**Right**	**Right**	**Right**	**Right**	**Right**
		TA_20%*_	TA_50%*_	TA_80%*_	**TA_MEMG20%†_**	**TA_MEMG50%†_**	**TA_MEMG80%†_**	**RF_MEMG20%†_**	**RF_MEMG50%†_**	**RF_MEMG80%†_**
	EA	0.30 (0.09)	0.58 (0.13)	0.76 (0.06)	1.05 (0.12)	1.29 (1.23)	3.01 (1.48)	1.00 (0.03)	1.03 (0.15)	1.73 (1.77)
	NA	0.27 (0.06)	0.54 (0.15)	0.80 (0.15)	1.01 (0.08)	1.05 (0.42)	1.26 (1.60)	1.01 (0.02)	1.00 (0.07)	1.01 (0.13)
**LE**		**Right**	**Right**	**Right**	**Left**	**Left**	**Left**	**Left**	**Left**	**Left**
		TA_20%*_	TA_50%*_	TA_80%*_	**TA_MEMG20%†_**	**TA_MEMG50%†_**	**TA_MEMG80%†_**	**RF_MEMG20%†_**	**RF_MEMG50%†_**	**RF_MEMG80%†_**
	EA	0.28 (0.14)	0.57 (0.18)	0.76 (0.11)	1.06 (0.33)	1.32 (1.23)	2.44 (1.42)	1.01 (0.02)	1.04 (0.08)	1.24 (1.46)
	NA	0.24 (0.08)	0.48 (0.21)	0.74 (0.11)	1.01 (0.10)	1.08 (0.75)	1.16 (1.16)	1.00 (0.01)	1.02 (0.04)	1.04 (0.44)

Values are median (interquartile range, IQR). UE, upper extremity; LE, lower extremity; NA, non-athletes; EA, endurance athletes; MVC, maximum voluntary contraction. *Voluntary mean EMG values are expressed as percentage of maximum voluntary contraction (MVC), 100% MVC = 1. ^†^Involuntary mean MEMG values are expressed as percent changes of baseline acitivity, value of 1 = no MEMG, value of *2* = 100% increase in MEMG compared to baseline activity.

EMG signal processing was performed offline using the software ProEMG (Motion Lab Systems Inc., Baton Rouge, LA, USA). EMG signals were rectified and the mean EMG activity was obtained, trial-by-trial, from all recorded muscles by estimation of root mean square values (100 ms) for each burst. The onset of each EMG burst of the main agonist of the active limb was defined as the time point when the mean EMG activity exceeded the baseline EMG-signal (BL, muscles at rest) by 3 standard deviations (baseline values + 3 standard deviations (SD)). The offset of each EMG burst was defined as the time point when the EMG signal fell below this value. EMG recordings from homologous muscles were time-locked to the on- and offset of the burst of the voluntary contracting muscle. Therefore, the temporal relationship between voluntary and involuntary muscle activity was preserved. Voluntary EMG amplitudes were normalized relative to the individual MVC. In accordance to previous investigations of MEMG (Sehm et al., 2010, 2016), MEMG amplitudes were expressed as the percentage change of 1 s pre-burst baseline EMG signal. Therefore a MEMG value of 1 means there was no change compared to the 1 s pre-burst baseline signal and a value of 2 means that the involuntarily occurring EMG activity increased by 100% compared to the 1 s pre-burst baseline signal. Prior to statistical analyses, all 25 trials of one force level were averaged for the voluntary and both distal and proximal involuntary muscles, respectively.

### Statistics

Between group comparisons (EA vs. NA) of maximal isometric force during MVC tests for UE and LE were performed using separate non-parametric Mann-Whitney-U-tests since data was not normally distributed. In addition, side comparisons of maximal voluntary force for UE and LE were performed within groups using Wilcoxon signed-rank-tests. For all statistical comparisons, a *p*-value of *p* < 0.05 was considered as significant.

Within group comparisons between voluntary mean activities for UE (left and right FDI) and LE (left and right TA) of all force levels (20%, 50%, 80% MVC) for EA and NA were performed using the non-parametric Friedman test of variance by ranks since the data was not normally distributed. *Post hoc* comparisons between force levels were performed using paired Wilcoxon signed-rank tests. Here, the significance level was Dunn-Bonferroni adjusted for three pairwise comparisons (20% vs. 50% MVC, 20% vs. 80% MVC, 50% vs. 80% MVC; *α*_adjusted_ = 0.0166). The same procedure was used to analyze the distal and proximal involuntary mean activities of UE (right and left FDI_MEMG_/BR_MEMG_) and LE (right and left TA_MEMG_/RF_MEMG_) respectively. Here, the significance level of *post hoc* Wilcoxon signed-rank tests was Dunn-Bonferroni adjusted for six pairwise comparisons (BL vs. 20% MVC, BL vs. 50% MVC, BL vs. 80% MVC, 20% vs. 50% MVC, 20% vs. 80% MVC, 50% vs. 80% MVC; *α*_adjusted_ = 0.0083).

Additional paired Wilcoxon signed-rank tests were used to perform side comparisons of the mean activities of voluntary and involuntary muscles within groups to look for possible effects of laterality.

Between group comparisons (EA vs. NA) of the mean voluntary and involuntary activities for each force level were performed using non-parametric paired Mann-Whitney-U-test with Bonferroni adjusted significance level (three comparisons per muscle, corrected *α* = 0.0167). Effect sizes were estimated with the Pearsons correlation coefficient (r) using *z*-values of *post hoc* performed Wilcoxon signed-rank tests divided by the root of the sample size (√N; Fritz et al., 2012).

## Results

There were no differences between EA and NA in terms of age, bodyweight or LQ (age: median EA: 23.00, NA: 25.00, *U* = 44.5, *p* = 0.300; bodyweight: median EA: 70.00, NA: 76, *U* = 40.0, *p* = 0.193; LQ: median EA: 100.00, NA: 84.00, *U* = 58.5, *p* = 0.898). As expected, EA had a significantly higher amount of weekly sports activities as compared to NA (median EA: 13.00, NA: 0.00, *U* = 0.00, *p* < 0.001).

For an overview of all obtained voluntary mean EMG and involuntary (MEMG) values (UE and LE) of NA and EA please refer to Table 1.

For statistical analyses and figures of secondary outcome measures (voluntary EMG values and proximal MEMG please see Supplementary Material).

### EMG and MEMG in Non-Athletes (NA)

#### Upper Extremity (UE)

The global Friedmann test revealed a significant effect for factor force level on baseline normalized MEMG in right FDI_MEMG_ ($\chi_{\left(3\right)}^{2}$ = 29.17, *p* < 0.001) and left FDI_MEMG_ ($\chi_{\left(3\right)}^{2}$ = 26.01, *p* < 0.001). *Post hoc* analyses showed significant MEMG in right FDI_MEMG_ for FDI_MEMG50%_ (*z* = −1.64, *p*_adjusted_ = 0.018, *r* = 0.49) and FDI_MEMG80%_ (*z* = −2.82, *p*_adjusted_ < 0.001, *r* = 0.85) as well as a significant difference in MEMG between right FDI_MEMG20%_ and FDI_MEMG80%_ (*z* = −2.00, *p*_adjusted_ < 0.001, *r* = 0.60). For left FDI_MEMG_ we found significant MEMG for left FDI_MEMG80%_ (*z* = −2.41, *p*_adjusted_ < 0.001, *r* = 0.73) as well as a significant difference in MEMG between left FDI_MEMG20%_ and FDI_MEMG80%_ (*z* = −2.32, *p*_adjusted_ < 0.001, *r* = 0.70). Side comparison of involuntary MEMG (within group comparison (NA)) between right and left FDI_MEMG_ failed to detect significant differences on all force levels (*p* > 0.05; *r* < 0.41 for all pairwise comparisons).

#### Lower Extremity (LE)

**Figure 2:**
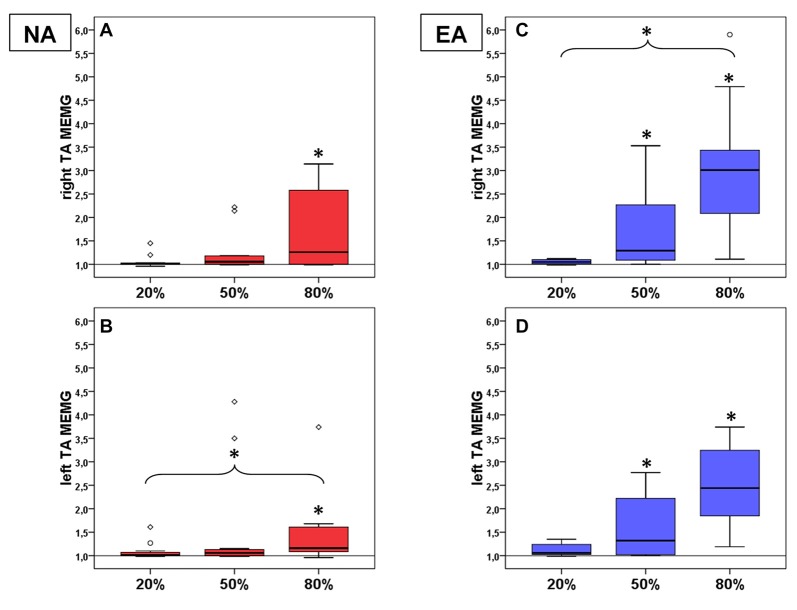
Mean MEMG values in TA for non-athletes (NA, *n* = 11, red boxes, right TA **(A)** left TA **(B)**) and endurance athletes (EA, *n* = 11, blue boxes, right TA **(C)** left TA **(D)**). All diagrams show the tested force levels (20%, 50%, 80% MVC) and involuntarily occuring mean MEMG values of left and right TA (expressed as percent changes of baseline signal, value of 1 = no MEMG, value of *2* = 100% increase in MEMG compared to baseline activity; * indicate significant changes compared to baseline or between force levels).

There also was a significant effect of the factor force level on baseline normalized MEMG (see Figures 2A,B) in right TA_MEMG_ ($\chi_{\left(3\right)}^{2}$ = 9.67, *p* = 0.022, *n* = 10) and left TA_MEMG_ ($\chi_{\left(3\right)}^{2}$ = 14.83, *p* = 0.002). *Post hoc* analyses showed significant MEMG in right TA_MEMG_ for TA_MEMG80%_ (*z* = −1.55, *p*_adjusted_ = 0.044, *r* = 0.49). For left TA_MEMG_ significant MEMG was identified for TA_MEMG80%_ (*z* = −1.96, *p*_adjusted_ = 0.002, *r* = 0.59) as well as a significant difference in MEMG betweenTA_MEMG20%_ and TA_MEMG80%_ (*z* = −1.59, *p*_adjusted_ = 0.023, *r* = 0.48). Together, our findings provide novel evidence for the existence of MEMG in distal homologous muscles (TA) in healthy participants. Again, side comparison of involuntary MEMG (within group comparison (NA)) between right and left TA_MEMG_ failed to detect significant differences on all force levels (*p* > 0.05; *r* < 0.20 for all pairwise comparisons).

### EMG and MEMG in Endurance Athletes (EA)

#### Upper Extremity (UE)

The global Friedmann test revealed a significant effect of the factor force level on baseline normalized MEMG in right FDI_MEMG_ ($\chi_{\left(3\right)}^{2}$ = 29.73, *p* < 0.001) and left FDI_MEMG_ ($\chi_{\left(3\right)}^{2}$ = 21.28, *p* < 0.001). *Post hoc* analyses showed significant MEMG in right FDI_MEMG_ for FDI_MEMG50%_ (*z* = −2.09, *p*_adjusted_ = 0.001, *r* = 0.63) and FDI_MEMG80%_ (*z* = −2.82, *p*_adjusted_ < 0.001, *r* = 0.85) as well as a significant difference in MEMG between FDI_MEMG20%_ and FDI_MEMG80%_ (*z* = −1.73, *p*_adjusted_ = 0.010, *r* = 0.52). For left FDI_MEMG_ we found significant MEMG for FDI_MEMG50%_ (*z* = −1.55, *p*_adjusted_ = 0.030, *r* = 0.47), FDI_MEMG80%_ (*z* = −2.36, *p*_adjusted_ < 0.001, *r* = 0.71) as well as a significant difference in MEMG between FDI_MEMG20%_ and FDI_MEMG80%_ (*z* = −1.73, *p*_adjusted_ = 0.010, *r* = 0.52). Side comparison of involuntary MEMG (within group comparison (EA)) between right and left FDI_MEMG_ failed to detect significant differences on all force levels (*p* > 0.05; *r* < 0.38 for all pairwise comparisons).

#### Lower Extremity (LE)

There also was a significant effect of the factor force level on baseline normalized MEMG (see Figures 2C,D) in right TA_MEMG_ ($\chi_{\left(3\right)}^{2}$ = 26.33, *p* < 0.001) and left TA_MEMG_ ($\chi_{\left(3\right)}^{2}$ = 21.67, *p* < 0.001). *Post hoc* analyses showed significant MEMG in right TA_MEMG_ for TA_MEMG50%_ (*z* = −1.91, *p*_adjusted_ = 0.003, *r* = 0.58), TA_MEMG80%_ (*z* = −2.50, *p*_adjusted_ < 0.001, *r* = 0.75) and a significant difference in MEMG between TA_MEMG20%_ and TA_MEMG80%_ (*z* = −1.82, *p*_adjusted_ = 0.006, *r* = 0.55). For left TA_MEMG_ significant MEMG was identified for TA_MEMG50%_ (*z* = −1.68, *p*_adjusted_ = 0.013, *r* = 0.51) and for TA_MEMG80%_ (*z* = −2.46, *p*_adjusted_ < 0.001, *r* = 0.74). Here, we again provide evidence for the existence of MEMG in distal homologous muscles (TA) of healthy athletes. Side comparison of involuntary MEMG (within group comparison (EA)) between right and left TA_MEMG_ again failed to detect significant differences on all force levels (*p* > 0.05; *r* < 0.20 for all pairwise comparisons).

### Comparison of EMG and MEMG between EA and NA

#### Upper Extremity (UE)

Comparing maximum isometric force values during MVC testing revealed no difference between EA and NA in left and right FDI (between group comparison (EA vs. NA) left FDI: median EA: 82.73, median NA: 82.41, *U* = 54.00, *p* = 0.699; right FDI: median EA: 83.89, median NA: 87.12, *U* = 46.00, *p* = 0.365). There were no significant differences between NA and EA in terms of voluntary mean EMG activities of left and right FDI (*p* > 0.0167; *r* < 0.16 for all pairwise comparisons). Furthermore no significant differences between NA and EA in terms of involuntary MEMG in right and left FDI_MEMG_ (*p* > 0.0167; *r* < 0.50 for all pairwise comparisons) were found.

#### Lower Extremity (LE)

**Figure 3:**
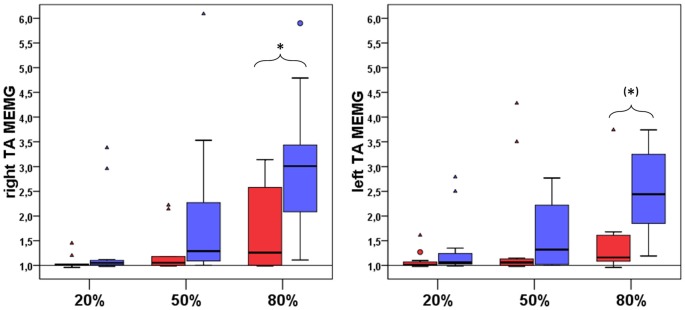
Group comparison of involuntary mean MEMG in right and left TA between NA (*n* = 11, red boxes) and EA (*n* = 11, blue boxes). All diagrams show the tested force levels (20%, 50%, 80% MVC) and involuntarily occuring mean MEMG values of right and left TA (expressed as percent changes of baseline signal, value of 1 = no MEMG, value of *2* = 100% increase in MEMG compared to baseline activity; * indicate a significant difference in the amount of MEMG between EA and NA, (*) indicate a statistical trend for a difference in the amount of MEMG between EA and NA).

Comparing maximum isometric force values during MVC testing revealed no difference between EA and NA in left and right TA (between group comparison (EA vs. NA) left TA: median EA: 249.52, median NA: 259.70, *U* = 60.00, *p* = 1.000; right TA: median EA: 286.82, median NA: 278.53, *U* = 55.00, *p* = 0.748). There were no significant differences between NA and EA in terms of voluntary mean-EMG-activities of left and right TA (*p* > 0.0167; *r* < 0.60 for all pairwise comparisons). Nevertheless significant differences between groups were found for involuntary MEMG (see Figure 3) of right TA_MEMG80%_ (between group comparison (NA vs. EA) NA: median = 1.26, EA: median = 3.01; M-W-U: *U* = 91.00, *p* = 0.01, *r* = 0.55). Additionally, group comparison for left TA_MEMG80%_ showed a strong statistical trend (between group comparison (NA vs. EA) NA: median = 1.16, EA: median = 2.44; M-W-U: *U* = 93.50, *p* = 0.08 (corrected Bonferroni *p*-value), *r* = 0.4).

## Discussion

Here we investigated the occurrence of physiological MEMG in healthy young individuals during voluntary unilateral isometric contractions of the upper (UE) and lower extremity (LE). In line with previous findings (Zijdewind and Kernell, 2001; Uttner et al., 2007; van Duinen et al., 2008; Sehm et al., 2010, 2016), we showed MEMG in distal and proximal homologous muscles during unmanual isometric contractions. Furthermore, our study provided novel quantitative evidence that physiological MEMG is also detectable during submaximal unilateral isometric contractions of leg muscles in healthy adults with the extent of MEMG increasing as a function of the exerted force. Group comparisons between EA and NA revealed that EA demonstrated a significant higher extent of MEMG specifically in the trained LE but only during strong isometric contractions of left TA (80% MVC). There was no difference in the extent of MEMG in UE between groups. Hence, the results indicate that long-term training might modulate the occurrence of MEMG in a training-specific manner, since LE muscles are predominantly trained under relatively high force demands in our EA sample in comparison to NA.

### Force-Dependent MEMG Modulation

The first evidence for physiological MEMG in LE indicates similar neural processing mechanisms for the emergence of involuntary activity in homologous muscles as previously described for the UE (Welniarz et al., 2015; Sehm et al., 2016). To date there are only few clinical studies describing the pathological form of MEMG in LE that lead to overt mirror movements in patients with Parkinson’s disease (Espay et al., 2005) and Tethered-Cord-syndrome (Tubbs et al., 2004). Contralateral co-contraction of leg muscles in healthy adults has previously been reported, but has not yet been systematically quantified (Dimitrijevic et al., 1992). Chiou et al. (2013b) provided first evidence that there is an increase in ipsilateral excitability of the leg representation within M1 during unilateral leg contractions. In contrast to the M1 hand representation during unimanual contractions (Chiou et al., 2013a) the observed increase in excitability for the leg representation area was not specifically restricted to homologous muscles. Therefore, the authors hypothesized divergent neural processing mechanisms between unilateral leg and hand contractions. One possible explanation could be found in the fundamentally different functional patterns of connectivity of UE (basic-mirror-movement-mode) in contrast to LE (alternating movement patterns) as a result of human evolutionary development (Chiou et al., 2013b). To date, however, these assumptions need to be supported by further scientific evidence.

In the present study, the observed MEMG showed its highest values in distal muscles and were significantly enhanced for 50% and 80% MVC (relative to baseline signal). This could be due to the fact that distal muscles (pinch-task: FDI; dorsiflexion of the foot: TA) represent the direct homologous counterpart to the main agonist of the performed motor tasks. Nevertheless, significant MEMG could also be observed in proximal muscles which did not directly contribute to the performance of the respective motor tasks, yet only for the highest force level (80% MVC, see Supplementary Material). This finding complements previous research, investigating MEMG of distal and proximal muscles during isometric wrist flexions (Sehm et al., 2010), whereby significant MEMG in proximal muscles could exclusively be detected under high force demands (70% MVC). Hence, it is reasonable to assume that the amount of synergistic homologous and heterologous co-contraction of muscles that are not directly involved in performing the motor task increases as a function of force demands. Even though the underlying mechanisms are still unknown, these co-contractions might assist force generation of the distal main mover through improved whole body stabilization (Dimitrijevic et al., 1992; Chiou et al., 2013b).

The extent of MEMG significantly increased in distal and proximal muscles of LE and UE respectively as a function of increased force demands. Again, this finding is in line with previous studies investigating MEMG in UE (Todor and Lazarus, 1986; Arányi and Rösler, 2002; Zijdewind et al., 2006; van Duinen et al., 2008; Sehm et al., 2010, 2016). Generally, it is assumed that there is an association between the amount of MEMG and the functional requirements of unilateral motor tasks. Beside force demands there are studies showing modulations of MEMG in response to central and peripheral fatigue caused by, *inter alia*, repetitive contractions (Liederman and Foley, 1987; Post et al., 2007, 2008; Cincotta and Ziemann, 2008), increased movement frequency (Uttner et al., 2007) as well as heightened cognitive load, for example visual stimuli provided simultaneously during task execution (Addamo et al., 2009).

Still, the question remains, what underlying mechanism modulates MEMG with regard to varying force demands? The most reasonable explanation seems to be the concept of motor overflow (Yensen, 1965; Todor and Lazarus, 1986; Perez and Cohen, 2008; Sehm et al., 2016). Progressively increasing force demands during unilateral wrist flexions have been shown to gradually decrease interhemispheric inhibition (IHI) between bilateral M1 (Perez and Cohen, 2008). This finding suggests that interhemispheric communication between both M1 during light unilateral contractions may be predominantly inhibitory. On the other hand, progressively stronger unilateral contractions reversed this effect into facilitation (IHF). Actually, there is experimental evidence for the occurrence of IHF in both hand (Ferbert et al., 1992; Hanajima et al., 2001; Baumer et al., 2006; Chiou et al., 2013a, 2014) and leg representation within M1 (Chiou et al., 2013b). Therefore, it appears reasonable to hypothesize that involuntary MEMG originates from bilateral M1 activation, caused by IHF (motor overflow) during unilateral contractions with high force demands.

In the present study, side comparisons during contractions of dominant or non-dominant extremities revealed no significant differences in MEMG. The existing literature contains mixed results regarding possible effects of laterality whereby higher MEMG during movements of the non-dominant (Armatas et al., 1996; Uttner et al., 2007) as well as dominant hand (Cernacek, 1961) have been described. However, studies which performed isometric contractions similar to our motor tasks have failed to show differences regarding the extent of MEMG (Hübers et al., 2008; Sehm et al., 2016). Hence, it is reasonable to hypothesize that the characteristics of task-dependent movement parameters, besides force requirements, might have an impact on possible side differences in observable MEMG (Cincotta and Ziemann, 2008).

### Training-Related MEMG Modulation: Comparison between Endurance Athletes (EA) and Non-Athletes (NA)

While there was no difference in absolute force values between EA and NA during MVC testing, we specifically demonstrated higher MEMG in the right TA muscle in EA (80% MVC). Furthermore, we found higher MEMG in right RF (80% MVC) in EA compared to NA.

The fact that absolute force values did not differ between groups seems to be surprising at first glance. However, this could be explained based on the fact that our motor tasks involved isolated muscles with small cross-sectional areas and therefore low ability to exert force. EA might have displayed higher force values during the execution of multi-joint compound movements like leg presses or squats as a result of improved intermuscular coordination between a multitude of contracting muscles due to their long-term exercise adaptations.

Additionally, EA and NA did not differ in MVC normalized mean EMG activity of voluntary contracting muscles in both extremities on all force levels. However, the differences in MEMG between groups, despite comparable force values and MVC normalized EMG activity of voluntary limbs, point towards differential neural processing mechanisms during unilateral contractions of LE. Interestingly, the difference in MEMG was specifically observable in LE. In this respect, we hypothesize that training-induced neuroplastic changes might be responsible for the observed findings since EA trained LE particularly as part of their respective sports discipline. This hypothesis is supported by the fact that we were not able to identify differences in the extent of MEMG between groups in UE. This might be due to the fact that participants in EA did not train their UE to a higher extent during daily practice compared to NA.

The increase in co-activation of ipsilateral M1 during unilateral contractions follows a homologous muscle-dependent effect (Chiou et al., 2013a, 2014). It is assumed that there is no general path for contralateral M1 to modulate the activation of ipsilateral M1. Instead, modulation from contralateral M1 to ipsilateral M1 appears to rely on the specific connection with the targeting muscle itself (Chiou et al., 2013a, 2014). Therefore, an increase in the integrity of transcallosal fibers, specifically those connecting leg motor areas due to neuroplastic changes induced by long-term endurance training could lead to the observed differences in the extent of MEMG in our study. Sports-related long-term practice and motor learning in general lead to functional and structural neuroplastic changes (Debarnot et al., 2014). Higher physical capacity in succession of moderate erobic training is able to alter mechanisms of interhemispheric communication (McGregor et al., 2015). Further, long-term unilateral strength training of leg muscles leads to an increase in ipsilateral (untrained) excitability of M1 as well as a decrease in IHI between bilateral M1 (Goodwill et al., 2012). Hence, it can be speculated that alterations in interhemispheric communication induced by long-term endurance training could provide an increased potential for a progressive reversal of IHI to IHF during strong unilateral contractions and therefore a lower threshold for the occurrence of motor overflow, which might be the reason for a greater extent of involuntary activity in athletes compared to NA. In support of this, Svatkova et al. (2015) specifically showed an increase in the integrity of motor relevant fibers passing through the CC (measured by fractional anisotropy—FA) induced by an erobic cycling regimen for several weeks. Indeed FA of transcallosal fibers correlated with the extent of ipsilateral facilitation of homologous muscle representation areas during unilateral contractions (Chiou et al., 2014). Furthermore, we have recently found evidence for an association between higher FA of motor-relevant transcallosal fibers connecting homologous muscle representation areas and the extent of individual MEMG in healthy untrained adults (Sehm et al., 2016). Based on these findings it can be speculated that trained individuals with greater structural integrity of motor-relevant transcallosal fiber tracts might exhibit more MEMG during strong unilateral contractions as well.

Further research is needed to confirm that sport-specific long-term training affects the amount of MEMG specifically in trained limbs. Therefore, future studies investigating different sports disciplines, for example strength sports (weightlifting, powerlifting) need to be conducted. Strength athletes perform movements with very high force demands using their LE as well as UE. As a consequence, one would assume significant differences in the extent of MEMG for both extremities compared to NA. In fact, a recent TMS study indirectly indicated this notion by showing an increase in excitability of ipsilateral (untrained) M1 as well as a decrease of IHI between bilateral M1 as a result of several weeks of unilateral hand strength training (Hortobagyi et al., 2011). These alterations in turn might possibly lead to greater MEMG in UE of these athletes.

The fact that MEMG in LE can be systematically detected in healthy adults opens a broad research field for future investigations. For example, future studies should provide deeper insights into the underlying neural mechanisms of MEMG by means of brain imaging techniques such as functional or structural MRI and/or non-invasive brain stimulation techniques such as TMS and transcranial direct current stimulation (tDCS). One limitation of the present study is that we are not able to further characterize underlying neurophysiological mechanisms of the phenomenon under investigation. In addition, using our study design, we cannot make direct inferences whether or not modulations in MEMG in EA were preexisting or indeed training-related. Therefore, future studies should perform specific training interventions and longitudinally investigate training-induced modulations of MEMG in a causative manner. To further support the assumption of exercise-induced limb-specific MEMG modulation, future studies investigating different sports disciplines with contrasting movement patterns and parameters should additionally be performed.

Future findings regarding the underlying neural mechanisms of MEMG in UE and LE could potentially illustrate facilitative adaptations in the context of sports performance, for example provide support for the hypothesis that bilateral co-contractions might assist force generation during strong muscular contractions through improved whole body stabilization (Dimitrijevic et al., 1992; Chiou et al., 2013b). In addition, neurophysiological insights could provide novel approaches for neural rehabilitation of movement disorder patients regarding optimal intervention protocols as well as attempts to modulate pathological interhemispheric communication through non-invasive brain stimulation techniques such as tDCS and TMS.

## Conclusion

We provide novel quantitative evidence that physiological MEMG is measurable in UE as well as LE during strong unilateral contractions in healthy adults. MEMG in both extremities significantly increases as a function of force demands. While the underlying neurophysiological mechanisms of MEMG still remain elusive, our study indicates, at least indirectly, that sport-related long-term training might affect the amount of MEMG during strong unilateral isometric contractions specifically in trained limbs.

## Author Contributions

All experiments were conducted at the Max Planck Institute for Human Cognitive and Brain Sciences Leipzig. TM, RK, PR and AV designed the study and experimental set-up. Participants were recruited and tested by TM and RK. TM, JL and CJS analyzed the data. All authors interpreted the data, contributed to the manuscript, reviewed it, approved the content of the final version and agree to be accountable for all aspects of the work. All persons designated as authors qualify for authorship, and all those who qualify for authorship are listed.

## Conflict of Interest Statement

The authors declare that the research was conducted in the absence of any commercial or financial relationships that could be construed as a potential conflict of interest.
