# Supplementary material for: Mirror Electromyografic Activity in the Upper and Lower Extremity: A Comparison between Endurance Athletes and Non-Athletes
Source: Front Hum Neurosci. 2017 Sep 29;11:485. doi: 10.3389/fnhum.2017.00485 (PMC5649197; doi:10.3389/fnhum.2017.00485)
Supplement: Supplementary file 1 [file Data_Sheet_1.docx]

Supplementary Material

**Mirror Electromyografic Activity in the Upper and Lower Extremity: A Comparison between Endurance Athletes and Non-Athletes**

**Tom Maudrich, Rouven Kenville, Jöran Lepsien, Arno Villringer, Patrick Ragert*^+^ and Christopher J. Steele^+^**

+ contributed equally

*** Correspondence:** Patrick Ragert: patrick.ragert@uni-leipzig.de

# Supplementary Results

**EMG and MEMG in non-athletes (NA)**

**Upper Extremity (UE)**

There was a significant side difference of maximal isometric force values during MVC testing between left and right FDI (within group comparison (NA): median left FDI = 82 N, median right FDI: 87 N, Wilcoxon-test: z = -2.40, p = 0.014, n = 11).

The factor force level showed a significant effect on MVC-normalized mean activities of left FDI (χ²(2) = 22.00, p< 0.001) and right FDI (χ²(2) = 22.00, p< 0.001). Post-hoc analyses for left FDI revealed a significant difference in mean activitiy between FDI_20%_ and FDI_80%_ (z = -2.00, p_adjusted_ < 0.001, r = 0.60). A similar significant post-hoc difference in mean activity could be revealed between right FDI_20%_ and FDI_80%_ (z = -2.00, p_adjusted_ < 0.001, r = 0.60). Side comparison of voluntary mean EMG activity between left and right FDI (within group comparison (NA)) showed no difference for all force levels (p > 0.05; r < 0.24 for all pairwise comparisons).

We also found a significant effect of the factor force level on proximal baseline normalized MEMG in right BR_MEMG_ (χ²(3) = 27.62, p< 0.001) and left BR_MEMG_ (χ²(3) = 22.57, p< 0.001). Post-hoc analyses showed significant MEMG in right BR_MEMG_ for BR_MEMG50%_ (z = -1.46, p_adjusted_ = 0.049, r = 0.45) and BR_MEMG80%_ (z = -2.77, p_adjusted_ < 0.001, r = 0.84) as well as a significant difference in the MEMG between BR_MEMG20%_ and BR_MEMG80%_ (z = -1.91, p_adjusted_ = 0.003, r = 0.58). For left BR_MEMG_ significant MEMG was identified for BR_MEMG80%_ (z = -2.41, p_adjusted_ < 0.001, r = 0.73) as well as a significant difference in MEMG between BR_MEMG20%_ and BR_MEMG80%_ (z = -1.86, p_adjusted_ = 0.004, r = 0.56). Side comparison of involuntary MEMG between right and left BR_MEMG_ again failed to detect significant differences on all force levels (p > 0.05; r < 0.35 for all pairwise comparisons).

**Lower Extremity (LE)**

Side comparisons of maximal isometric force during MVC testing failed to detect a difference between left and right TA (within group comparison (NA): median left TA = 260 N, Median right TA: 279 N , Wilcoxon-test: z = -1.33, p = 0.206, n = 11).

The factor force level showed a significant effect on MVC normalized mean activities (see Fig. S1, (A) and (B)) of left TA (χ²(2) = 20.00, p< 0.001, n = 10) and right TA (χ²(2) = 22.00, p< 0.001). Post-hoc analyses for left TA revealed a significant difference in mean activitiy between TA_20%_ and TA_80%_ (z = -2.00, p_adjusted_ < 0.001, r = 0.60). A similar significant post-hoc difference in mean activity could be revealed between TA_20%_ and TA_80%_ (z = -2.00, p_adjusted_ < 0.001, r = 0.60). Again, side comparison of voluntary mean EMG activity between left and right TA (within group comparison (NA)) showed no difference for all force levels (p > 0.05; r < 0.24 for all pairwise comparisons).

In terms of proximal involuntary activity we could not find significant MEMG (see Fig. S2, (A) and (B)) either in right RF_MEMG_ nor left RF_MEMG_ (p > 0.05; r < 0.51 for all pairwise comparisons). Furthermore side comparison of involuntary MEMG between right and left RF_MEMG_ failed to detect significant differences on all force levels (p > 0.05; r < 0.45 for all pairwise comparisons).

**EMG and MEMG in endurance athletes (EA)**

**Upper Extremity (UE)**

Side comparisons of maximal isometric force during MVC testing failed to detect a difference between left FDI and right FDI (within group comparison (EA): median left FDI = 83 N, median right FDI: 84 N, Wilcoxon-test: z = -0.18, p = 0.898).

The factor force level showed a significant effect on MVC normalized mean activities of left FDI (χ²(2) = 22.00, p< 0.001) and right FDI (χ²(2) = 22.00, p< 0.001). Post-hoc analyses for left FDI revealed a significant difference in mean activitiy between FDI_20%_ and FDI_80%_ (z = -2.00, p_adjusted_ < 0.001, r = 0.60). A similar significant post-hoc difference in mean activity could be revealed between right FDI_20%_ and FDI_80%_ (z = -2.00, p_adjusted_ < 0.001, r = 0.60). Side comparison of voluntary mean EMG activity between left and right FDI (within group comparison (EA)) showed no difference for all force levels (p > 0.05; r < 0.06 for all pairwise comparisons).

We also found a significant effect of the factor force level on proximal baseline normalized MEMG in right BR_MEMG_ (χ²(3) = 31.62, p< 0.001) and left BR_MEMG_ (χ²(3) = 25.00, p< 0.001). Post-hoc analyses showed significant MEMG in right BR_MEMG_ for BR_MEMG50%_ (z = -2.05, p_adjusted_ = 0.001, r = 0.62) and BR_MEMG80%_ (z = -2.86, p_adjusted_ < 0.001, r = 0.86) as well as a significant difference in MEMG between BR_MEMG20%_ and BR_MEMG80%_ (z = -1.96, p_adjusted_ = 0.002, r = 0.59). For left BR_MEMG_ significant MEMG was found for BR_MEMG80%_ (z = -2.46, p_adjusted_ < 0.001, r = 0.74) as well as a significant difference in MEMG between BR_MEMG20%_ and BR_MEMG80%_ (z = -2.18, p_adjusted_ < 0.001, r = 0.66).Furthermore side comparison of involuntary MEMG between right and left BR_MEMG_ failed to detect significant differences on all force levels (p > 0.05; r < 0.34 for all pairwise comparisons).

**Lower Extremity (LE)**

Side comparisons of maximal isometric force during MVC testing showed no difference between left TA and right TA (within group comparison (EA): median left TA = 250 N, median right TA: 287 N, Wilcoxon-test: z = -0.45, p = 0.700, n = 11).

The factor force level showed a significant effect on MVC normalized mean activities (see Fig. S1, (C) and (D)) of left TA (χ²(2) = 22.00, p< 0.001) and right TA (χ²(2) = 22.00, p< 0.001). Post-hoc analyses for left TA revealed a significant difference in mean activitiy between TA_20%_ and TA_80%_ (z = -2.00, p_adjusted_ < 0.001, r = 0.60). A similar significant post-hoc difference in mean activity was found between right TA_20%_ and TA_80%_ (z = -2.00, p_adjusted_ < 0.001, r = 0.60). Again, side comparison of voluntary mean EMG activity between left and right TA (within group comparison (EA)) showed no difference for all force levels (p > 0.05; r < 0.20 for all pairwise comparisons).

In contrast to NA we also found a significant effect of the factor force level on proximal baseline normalized MEMG (see Fig. S2, (C) and (D)) in right RF_MEMG_ (χ²(3) = 18.71, p< 0.001) and left RF_MEMG_ (χ²(3) = 20.21, p< 0.001). Post-hoc analyses showed significant MEMG in right RF_MEMG_ for RF_MEMG80%_ (z = -1.82, p_adjusted_ = 0.006, r = 0.55) as well as a significant difference in MEMG between RF_MEMG20%_ and RF_MEMG80%_ (z = -2.09, p_adjusted_ = 0.001, r = 0.63) and between RF_MEMG50%_ and RF_MEMG80%_ (z = -1.55, p_adjusted_ = 0.030, r = 0.48). For left RF_MEMG_ significant MEMG was found for RF_MEMG80%_ (z = -2.27, p_adjusted_ < 0.001, r = 0.68) as well as a significant difference in MEMG between RF_MEMG20%_ and RF_MEMG80%_ (z = -1.72, p_adjusted_ = 0.010, r = 0.52). Together, our findings also provide novel evidence for the existence of MEMG in proximal muscles (RF) in healthy athletes. Furthermore side comparison of involuntary MEMG between right and left TA_MEMG_ failed to detect significant differences on all force levels (p > 0.05; r < 0.29 for all pairwise comparisons).

**Comparison of EMG and MEMG between EA and NA**

**Upper Extremity (UE)**

Groups also did not show significant differences in involuntary MEMG in right and left BR_MEMG_ (p > 0.0167; r < 0.65 for all pairwise comparisons).

**Lower Extremity (LE)**

Furthermore significant differences between groups were identified for involuntary MEMG (see Fig. S3) of right RF_MEMG80%_ (between group comparison (NA vs. EA) NA: median = 1.01, EA: median = 1.73; M-W-U: U = 91.50, p = 0.008, r = 0.78).

#
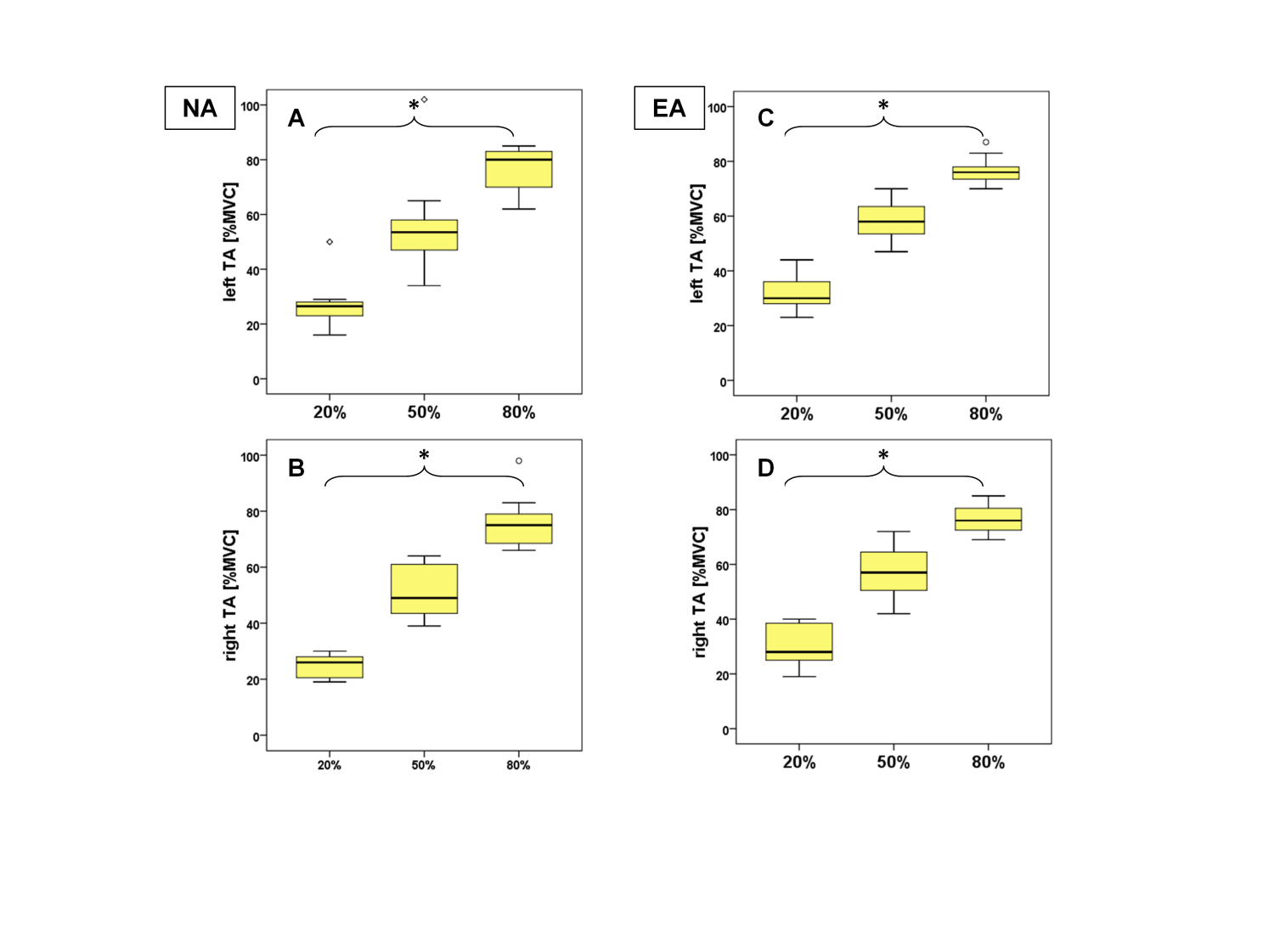
Supplementary Figures

**Figure S1. Mean voluntary EMG values in TA for non-athletes (NA) and endurance athletes (EA).** All diagrams show the tested force levels (20%, 50%, 80% MVC) and mean EMG values of right and left TA (NA = **(A)** and **(B)**; EA = **(C)** and **(D)**) during voluntary unilateral contractions (expressed as percentage of maximum voluntary contraction (MVC), 100 = 100% MVC; * indicate significant differences between force levels).


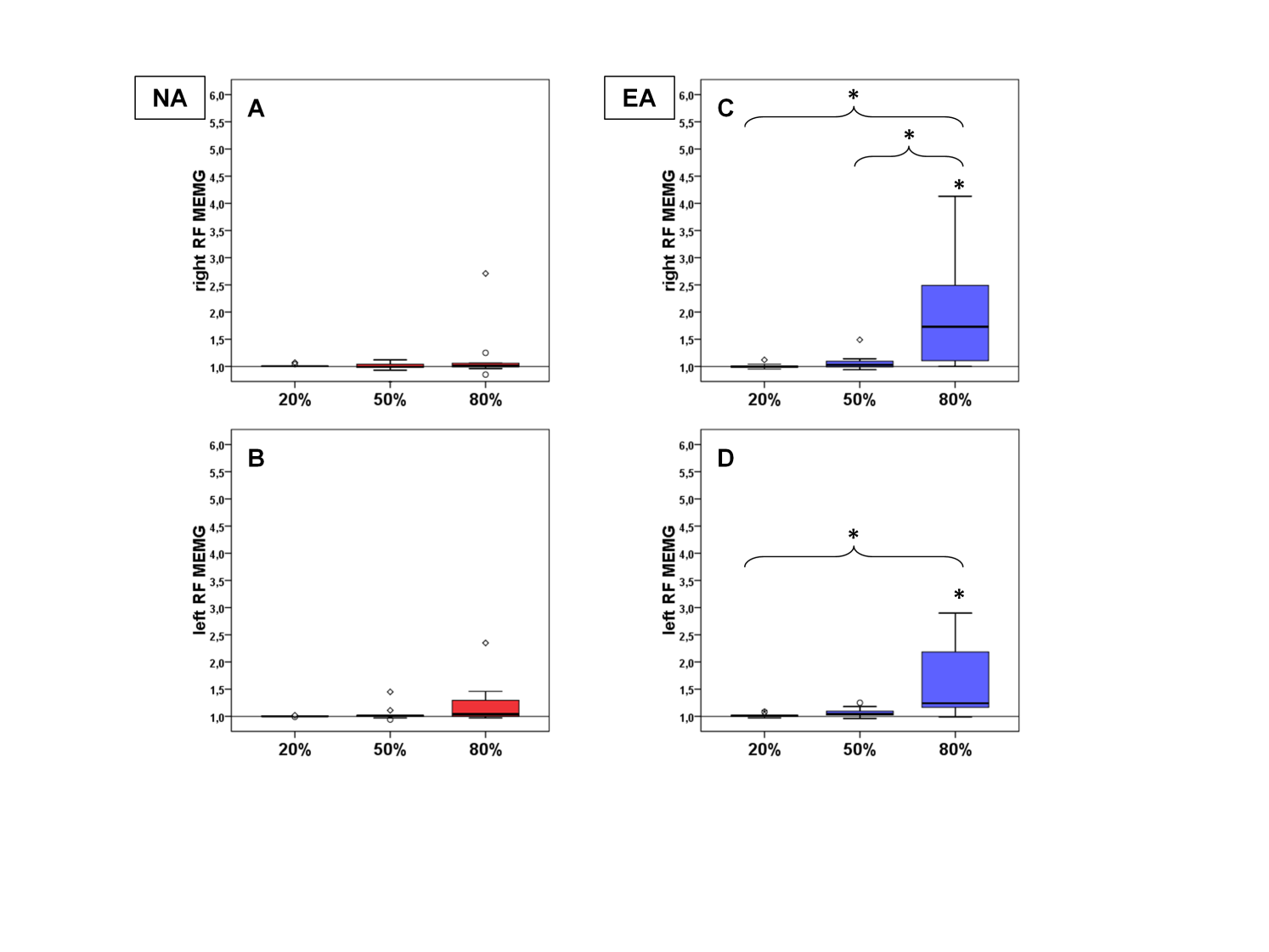


**Figure S2. Mean MEMG values in RF** for non-athletes (NA, n = 11, red boxes, right RF **(A)**, left RF **(B)**) and endurance athletes (EA, n = 11, blue boxes, right RF **(C)**, left RF **(D)**). All diagrams show the tested force levels (20%, 50%, 80% MVC) and involuntarily occuring mean MEMG of left and right RF TA (expressed as percent changes of baseline signal, value of 1 = no MEMG, value of 2 = 100% increase in MEMG compared to baseline activity; * indicate significant changes compared to baseline or between force levels).


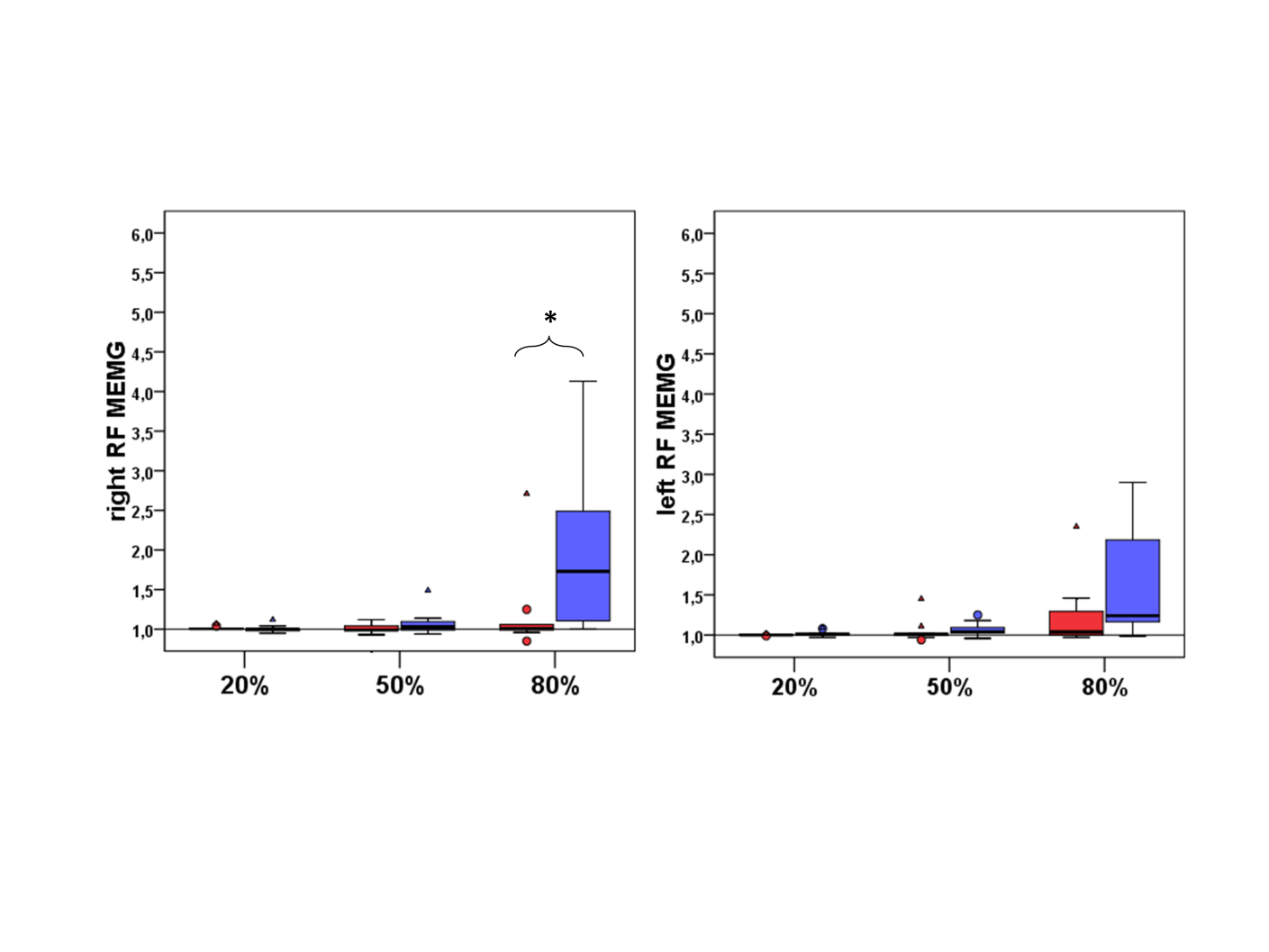


**Figure S3. Group comparison of involuntary mean MEMG values in right and left RF** between non-athletes (NA, n = 11, red boxes) and endurance athletes (EA, n = 11, blue boxes). All diagrams show the tested force levels (20%, 50%, 80% MVC) and involuntarily occuring mean MEMG values of left and right RF (expressed as percent changes of baseline signal, value of 1 = no MEMG, value of 2 = 100% increase in MEMG compared to baseline activity; * indicate a significant difference in the amount of MEMG between EA and NA).
